# Tackling the Problem of Sensing Commonly Abused Drugs Through Nanomaterials and (Bio)Recognition Approaches

**DOI:** 10.3389/fchem.2020.561638

**Published:** 2020-11-04

**Authors:** Florina Truta, Anca Florea, Andreea Cernat, Mihaela Tertis, Oana Hosu, Karolien de Wael, Cecilia Cristea

**Affiliations:** ^1^Department of Analytical Chemistry, “Iuliu Haţieganu” University of Medicine and Pharmacy, Cluj-Napoca, Romania; ^2^Antwerp X-ray Analysis, Electrochemistry and Speciation Research Group, University of Antwerp, Antwerp, Belgium; ^3^NANOlab Center of Excellence, University of Antwerp, Antwerp, Belgium

**Keywords:** electrochemical sensors, nanomaterials, biomimetics, illicite drugs, powders, biological fluids, wastewaters

## Abstract

We summarize herein the literature in the last decade, involving the use of nanomaterials and various (bio)recognition elements, such as antibodies, aptamers and molecularly imprinted polymers, for the development of sensitive and selective (bio)sensors for illicit drugs with a focus on electrochemical transduction systems. The use and abuse of illicit drugs remains an increasing challenge for worldwide authorities and, therefore, it is important to have accurate methods to detect them in seized samples, biological fluids and wastewaters. They are recently classified as the latest group of “emerging pollutants,” as their consumption has increased tremendously in recent years. Nanomaterials, antibodies, aptamers and molecularly imprinted polymers have gained much attention over the last decade in the development of (bio)sensors for a myriad of applications. The applicability of these (nano)materials, functionalized or not, has significantly increased, and are therefore highly suitable for use in the detection of drugs. Lately, such functionalized nanoscale materials have assisted in the detection of illicit drugs fingerprints, providing large surface area, functional groups and unique properties that facilitate sensitive and selective sensing. The review discusses the types of commonly abused drugs and their toxicological implications, classification of functionalized nanomaterials (graphene, carbon nanotubes), their fabrication, and their application on real samples in different fields of forensic science. Biosensors for drugs of abuse from the last decade's literature are then exemplified. It also offers insights into the prospects and challenges of bringing the functionalized nanobased technology to the end user in the laboratories or in-field.

## Introduction

Despite the “war on drugs,” drug abuse is a major concern worldwide with devastating effects on human health, economy and communities. Cannabis is one of the longest-established drugs in Europe and is the most commonly used illicit drug, with nearly 20% of those in the 15–24 age group reporting having used cannabis in the last year. In 2017, 1.1 million drug seizures were reported in Europe. Herbal cannabis accounts for 42% of total number of drug seizures in 2017, followed by cannabis resin with 28%. Cocaine is next on the list with 10%, followed by amphetamines 5%, heroin 4%, and MDMA 3% (European Monitoring Centre for Drugs and Drug Addiction, 2019).

The current detection of illicit drugs in seized street samples is performed by quick, presumptive tests, such as color tests, however these tests lack selectivity, and need further confirmation by expensive and time-consuming chromatography-mass spectrometry (GC-MS) methods. Therefore, it is of interest to develop new sensing technologies that allow a fast, sensitive, and selective detection of illicit drugs in-the-field.

Electrochemical methods proved to be a great alternative for the fast determination of drugs with high sensitivity and selectivity and are easy to miniaturize into portable devices to be used in-the-field. Electrochemical detection of several illicit drugs has been reported in literature and proved to be effective for accurately detecting illicit drugs in complex adulterated street samples (Florea et al., 2019c). Nanomaterials and biomimetic platforms (e.g., aptamers, molecularly imprinted polymers-MIP) have gained much attention over the last decade in the development of (bio)sensors for a myriad of applications. The applicability of these (nano)materials makes them highly suitable for use in the detection of drugs of abuse. Lately, (functionalized) nanomaterials have assisted in the detection of illicit drugs fingerprints, providing large surface areas, rich functional groups and unique properties that facilitate sensitive and selective sensing (Zhang et al., 2017; Rawtani et al., 2019).

Current drug measurements in body fluids, such as blood, urine, and saliva, are performed by centralized laboratories using classical methods and bulky instruments based on liquid chromatography, GC-MS and GC-MS with solid phase micro-extraction (Ya et al., 2015; Purschke et al., 2016). Electrochemical methods are also suitable for the detection of drugs of abuse in complex matrices such as body fluids. Since drugs are present in low concentrations in body fluids the integration of nanomaterials in electrochemical sensors is advantageous. Saliva-based drug detection is of particular interest for on-site screening, as it is a non-invasive method, and unlike blood assays, does not need an invasive sample collection (Huestis and Smith, 2018). Most drugs degrade to their metabolites and are therefore found along with their metabolites in body fluids. Sometimes special attention in handling these samples is required. For example, heroin degrades to 6-monoacetylmorphine and morphine *in vitro* and *in vivo*. *In vitro* the degradation is also dependent on the pH and temperature. Analytical methods that include quantification of heroin recognize that heroin and 6-monoacetylmorphine are unstable in certain matrices and suggest using freshly prepared solutions (Jones et al., 2013).

This review focuses on recent development of (bio)sensors for the detection of drugs of abuse in seized street samples and biological fluids. Given the importance of drug metabolites for the detection in body fluids aspects regarding pharmacokinetics and toxicology of common drugs of abuse are briefly discussed. The integration of nanomaterials and affinity elements, such as aptamers and MIP into the sensors for increased sensitivity and selectivity is presented. Finally, further improvements and the necessity to tackle the problem of selective detection are discussed.

## Types of Drugs of Abuse and Their Toxicological Implications

An overall increase in drug-related deaths has been observed over the last 5 years, with increases reported in all age groups above the age of 30 years. Drug overdose deaths are rarely associated with the consumption of one substance alone. Modern drug consumption patterns are highly dynamic, with an increased number of drugs appearing on the market (European Monitoring Centre for Drugs and Drug Addiction, 2019).

According to the European Drug Report 2019 cannabis is the most used drug with 24.7 million users aged 15–64 in 2018 (European Monitoring Centre for Drugs and Drug Addiction, 2019). Δ^9^-tetrahydrocannabinol (Δ-THC) is the compound responsible for the psychoactive actions of the drug. Besides Δ^9^-THC, organic cannabis products contain additional cannabinoids which do not produce psychoactive effects, such as cannabinol, and cannabidiol (Rhee et al., 1997; Pertwee, 2005). Synthetic cannabinoids are compounds prepared by chemical synthesis to produce the same effects as Δ^9^-THC and have emerged more and more recently. They are more unsafe, as their effects are more potent, and they also contain unknown chemicals made-up with the drug that may have negative effects on human health. Δ^9^-THC metabolite, THC-COOH, can be detected in body fluids, such as saliva, urine and blood, three to six hours after its consumption and can be retained in the body for several days (Huestis, 2007). Detection in saliva would be preferred, as it is non-invasive and simple compared to detection in blood. The concentration of Δ^9^-THC in saliva is a function of the time since the cannabis consumption (Dobri et al., 2019), with a maximum salivary Δ^9^-THC level of 16 μmol *L*^−1^ observed 1–2 h after consuming the drug (Niedbala et al., 2001). Acute cannabis intoxication is associated with impaired driving, significantly increasing the odds of motor vehicle collision (Dahlgren et al., 2020). The use of cannabis is associated with pathological and behavioral toxicity (Huestis, 2007). Effects of short-term use include impaired short-term memory, altered attention, hallucinations and distortions of spatial perception, anxiety, bronchodilatation, palpitations, increased heart rate, nausea, and appetite change (Volkow et al., 2014; Cohen and Weinstein, 2018). Chronic effects of cannabis and cannabinoids use include long-lasting cognitive impairments and a risk for developing mental disorders (anxiety, depression, bipolar disorder, schizophrenia) (Degenhardt and Hall, 2008; Volkow et al., 2014; Cohen and Weinstein, 2018).

Cocaine is the second most prevalent illicit drug with 3.9 million users in 2018 and an increasing number and volume of seizures. Cocaine is a stimulant type drug which appears as white powder, paste or rock-like and is often adulterated with compounds with similar effects (e.g., procaine, having anesthetic effects like cocaine, or diluted with non-active compounds, e.g., starch, glucose, talcum. Cocaine is used by smoking, snorting, or injecting, for effects of euphoria, elevated mood, and increased energy).

**Figure 1:**
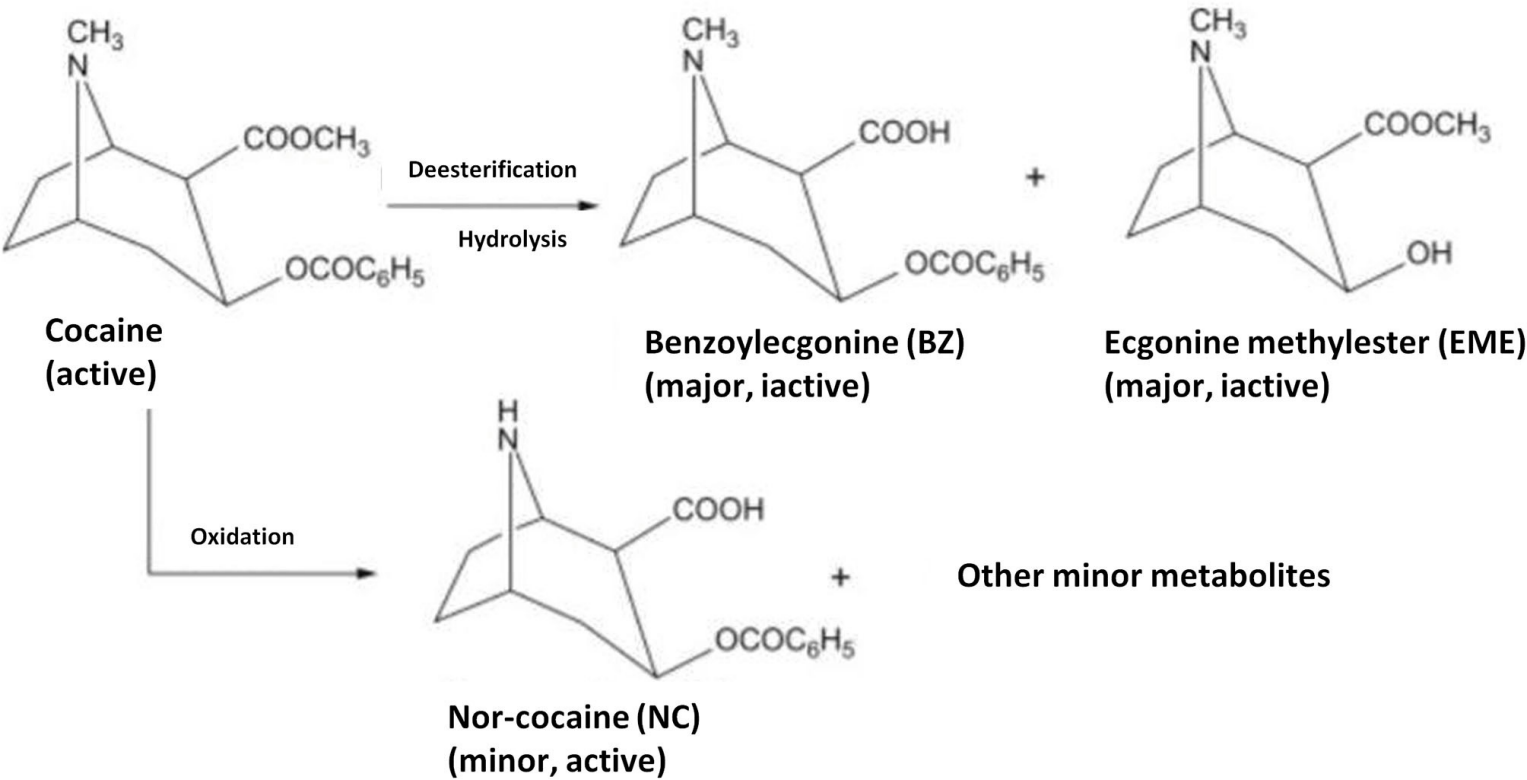
Metabolism of cocaine to major inactive metabolites, benzoylecgonine(BZ) and ecgonine methylester (EME), and to a minor active metabolite nor-cocaine (NC). Reproduced from Shimomura et al. (2019).

Cocaine is metabolized mainly to benzoylecgonine and in lesser amounts to ecgonine methyl ester, as well as to norcocaine, p-hydroxycocaine, m-hydroxycocaine, p-hydroxybenzoylecgonine, and m-hydroxybenzoylecgonine. The detailed main mechanism is presented in Figure 1 (Munoz et al., 2011; Baciu et al., 2015).

Cocaine and its metabolites can be detected in body fluids such as saliva, urine, and blood, and are accumulated in hair. Urine testing is common for monitoring cocaine users in drug treatment. Peak urine cocaine concentrations after snorting is of 300–24,000 ng *mL*^−1^ in <1 h, while peak urine benzoylecgonine (a metabolite of cocaine) concentrations ranged from 8,100 to 70,800 ng *mL*^−1^ after of 4–8 h (Huestis et al., 2007). Cocaine concentration in hair is ≥ 0.5 ng *mg*^−1^, while benzoylecgonine concentration reaches ≥ 0.05 ng *mg*^−1^. Acute effects of cocaine use include increased heart rate and blood pressure, nausea and appetite change, vertigo, and tremor. Chronic use leads to severe constant fatigue, problems with memory, attention, strong headaches, severe weight loss, motor, and sexual disfunction (Cregler, 1988).

Amphetamine type stimulants are a class of drugs mainly referring to amphetamine and methamphetamine, but other drugs are also included in this group such as methylenedioxymethamphetamine (MDMA) (ecstasy), 3,4-methylenedioxy-N-ethylamphetamine (MDEA), methcathinone or ephedrine. As the name suggests this type of drugs has stimulant effects on the nervous system. MDMA is the third most prevalent drug of abuse with 2.6 million users in 2018, while amphetamine is the fourth most used with 1.7 million users in 2018 (European Monitoring Centre for Drugs and Drug Addiction, 2019). Amphetamine type stimulants have been used as medicines for the stimulant and appetite reducing effects and are still used today to treat narcolepsy or attention deficit disorder. Short-term use effects include agitation, alertness, anxiety, increased breath and heart rate, stomach pain, dilated pupils, and blurred vision. Long term use include sleeping problems, depression and chronic anxiety, poor memory, high blood pressure, severe weight loss, and risk of lung diseases (Heal et al., 2013; Cao et al., 2016; Haj-Mirzaian et al., 2018).

Despite the decreasing use in Europe in the last year, as well as the decrease of HIV cases associated with injecting heroin, opioids' use continues to make a major contribution to the health and social costs in Europe, and the threats posed by this class of drug may even be growing. The Drug Report 2019 accounts 1.3 million high-risk opioid users (European Monitoring Centre for Drugs and Drug Addiction, 2019). Among the most used opioids currently are synthetic opioids, particularly fentanyl derivatives. For example, carfentanyl is a very potent opioid that leads to severe poisoning and deaths, which is trafficked in very small amounts making it difficult to detect. An increasing role is played also by synthetic opioids that are usually used as medicines for (e.g., pain relief). When it comes to seizures, heroin is the most common opioid drug on the European market. Heroin appears as a white powder and can be mixed with other white powders to dilute the sample such as paracetamol, quinine, sugars, or powdered milk. Adulterated heroin samples appear as white to yellowish or brownish color. Black tar is another form of heroin which appears as a black sticky- or coal-like substance. Regarding heroin pharmacokinetics, heroin degrades rapidly to 6-monoacetylmorphine and morphine *in vivo* and *in vitro*, thus when analyzed in body fluids this should be taken into consideration. Heroin and 6-monoacetylmorphine are found in saliva 2 min after smoke or intravenous administration. The levels in body fluids of heroine metabolites, morphine and 6-monoacetylmorphine, are in the nM range. The rate of degradation depends on the type of biological sample and the duration and conditions of storage. The degradation *in vitro* is also dependent on the pH and temperature (Jones et al., 2013). Acute effects of heroin consumption include a “rush” of pleasurable feelings followed by flushing of the skin, reduced breathing and heart rate and severe itching. Chronic use may cause inflammation of the gums, decreased memory and intellectual capacity, insomnia, impotence, infection of the blood vessels, muscle weakness and pain and strong physical dependence and tolerance (Jones et al., 2013).

## Examples of (BIO)Sensors for Drugs of Abuse

**Figure 2:**
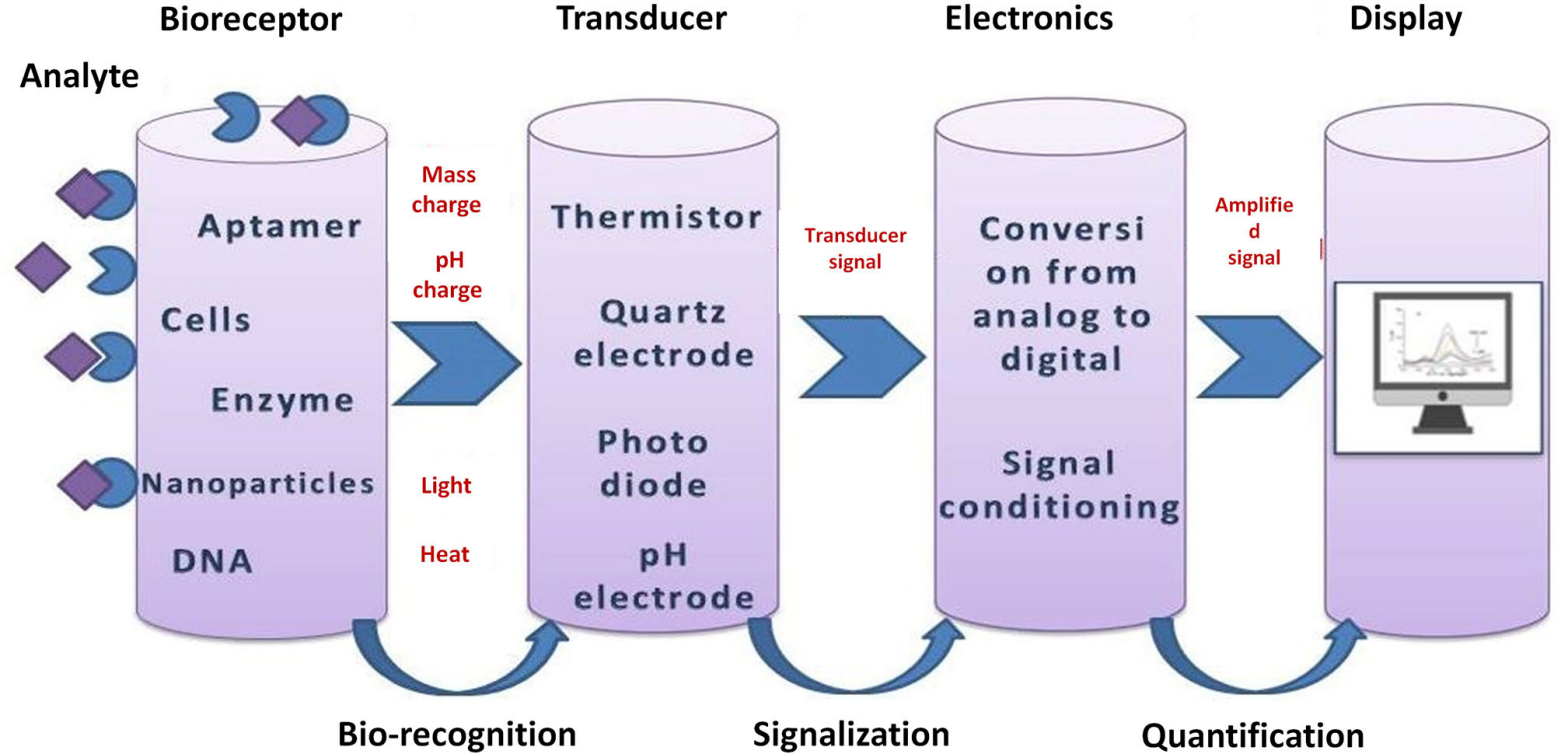
Schematic representation of a (bio)sensor.

Nowadays, (bio)sensors are widely used in biomedical diagnosis, point-of-care monitoring of treatment and disease progression, but also in other areas such as environmental monitoring, food control, drug discovery, forensics, and biomedical research. Usually (bio)sensors are coupled with high-affinity biomolecules or other recognition elements, such as molecularly imprinted polymers, and in this way can allow the sensitive and selective detection of a high range of analytes. A typical (bio)sensor is represented in Figure 2. A (bio)sensor is composed from the following units: an analyte (the substance of interest), a (bio)receptor (a molecule that specifically recognizes the analyte), a transducer (an element that converts one form of energy into another), electronics (processes the transducer signal and prepares it for display) and a display (Bhalla et al., 2016). The advantages of (bio)sensing systems are the possibility of miniaturization and automation, easy fabrication and modification, low cost, and sensitivity.

Our review provides the most relevant improvements and innovations in sensors and biosensors development for the analysis of a range of commonly-encountered illicit substances in different types of matrices, such as seized materials, biological, and environmental samples. The targets we selected refer to stimulants, such as amphetamine type drugs, cathinone and cocaine, depressants, such as opium-related compounds and cannabinoids.

The most relevant studies in the literature of the lasts 10 years about (bio)sensors developments are summarized in Table 1.

**Table 1:** Overview list of (bio)sensors for detection of abused drugs.

**Classes of drugs of abuse**	**Target drugs**	**Type of (bio) sensor**	**Pretreatment**	**Detection analytical method**	**Limit of detection (LOD) or limit of quantification (LOQ)**	**Samples**	**References**
Narcotics	Morphine	RGO-Pd	-	DPV	LOQ = 0.34 μmol *L*^−1^ LOQ = 14 μmol *L*^−1^ LOD = 0.012 μmol *L*^−1^	Human urine	Atta et al., 2014
		EGO-modified SPE	-	DPV	LOD = 8.77^*^10^−6^ μmol *mL*^−1^	Urine sample	Maccaferri et al., 2019
		IL/NiO/CNTCPE	-	SWV	LOD = 0.01 μmol *L*^−1^ LOQ = 1.63 μmol *L*^−1^	Human urine, pharmaceutical samples	Sanati et al., 2014
		GNSs modified GC electrode	-	DPV	LOQ = 65 μmol *L*^−1^ LOD = 0.4 μmol *L*^−1^		Navaee et al., 2012
	Heroine	GNSs modified GC electrode	-	DPV	LOQ = 100 μmol *L*^−1^ LOD = 0.5 μmol *L*^−1^		Navaee et al., 2012
Stimulants	Cocaine	Supramolecular aptasensor on supramolecular aptamer, rolling circle amplification combined with multiplex binding of the biotin-strepavidin system for cocaine detection.	The aptamer fragments were assembled to a supramolecular aptamer, in the presence of cocaine, conjugates to streptavidin for anchoring of biotinylated circular DNA. These modifications initiates RCA and enables sensitive electrochemical-enzymatic readout.	DPV	LOD = 0.0013 μmol *L*^−1^ (at *S/N*=3) LOQ = 0.002 μmol *L*^−1^	Spiked urine samples	Shen et al., 2015
		SPCEs MWCNTs-SPEs	-	SWV	LOQ = 10 μmol *L*^−1^	Street samples	Asturias-Arribas et al., 2014
		Aptasensor platform	Electrodeposition of thiophene macromonomer bearing polypeptides.	DPV		Synthetic biological fluids (urine and saliva)	Bozokalfa et al., 2016
		Pt-SPEs	Pt-SPEs was covered COHCFe	CV	LOD = 28.8 μmol *L*^−1^ LOQ = 96.2 μmol *L*^−1^	Seized samples	Balbino et al., 2016
		BDDE	-	BIA-SWV	LOQ = 0.198 μmol *L*^−1^ LOD = 0.89 μmol *L*^−1^	-	Freitas et al., 2017
		GSPE		SWV	LOD = 3 μmol *L*^−1^ LOQ = 10 μmol *L*^−1^	Street samples	De Jong et al., 2018a
		GPH-SPE	The electrodeposition of PABA and OPD	SWV	LOQ = 50 μmol *L*^−1^ LOQ = 100 μmol *L*^−1^	-	Florea et al., 2018
		Ultrasensitive electrochemical nanoaptasensor	1. Deposition of AuNPs on the surface of GCE 2. Covalent attachment of Apt	DPV	-	Serum	Roushani and Shahdost-Fard, 2016
		Potentiometric sensor based on nanoMIPs	-	-	LOQ = 0.001 μmol *L*^−1^	Blood serum sample	Smolinska-Kempisty et al., 2017
	Methcatinone	SPEs	-	CV	LOQ = 0.098 μmol *L*^−1^ (at pH=12) LOD = 0.273 μmol *L*^−1^	-	Smith et al., 2013
	Mephedrone	SPEs	-	CV	LOQ = 0.09 μmol *L*^−1^ (at pH=2) LOD = 0.224 μmol *L*^−1^	-	Smith et al., 2013
	MEC	SPEs	-	CV	LOQ = 0.083 μmol *L*^−1^ (at pH=2) LOD = 0.44 μmol *L*^−1^	-	Smith et al., 2013
	Methcathinone	MIF	-	DPV	LOD = 0.0202 nmol *mL*^−1^	Serum samples	Zang et al., 2013
	Cathinone	MIF	-	DPV	LOD = 0.059 nmol *mL*^−1^	Serum samples	Zang et al., 2013
		-	-				
	MDMA	EPAD coated with ZnONRs	-	CV	LOQ= 1 μmol *L*^−1^ LOD=0.1 μmol *L*^−1^	Human saliva, sweat, and urine.	Narang et al., 2018
		SPEs BDD GC	-	DPV	LOD = 0.207 μmol *mL*^−1^	-	Cumba et al., 2016
	PMA	SPEs BDD GC	-	DPV	LOD = 0.048 μmol *L*^−1^	-	Cumba et al., 2016
	MDMA/PMA	SPEs BDD GC	-	DPV	LOD = 1.29/0.227 μmol *L*^−1^	-	Cumba et al., 2016
Cannabis	SCs		-		-		
				-			
Δ^9^ −THC		SPE with NAMM	-	CA	LOQ = 79.64 nmol *mL*^−1^	Saliva	Wanklyn et al., 2016
		GC disk electrode	30 s pre-concentration step under an applied potential of ^−1^.2 V The elimination of chemical interferences from samples was achieved through prior purification using the TLC technique	CV	LOD = 1.08 nmol *mL*^−1^ LOQ = 7.64 nmol *mL*^−1^	Hamp and hashish confiscated by the police	Balbino et al., 2012
		C-SPEs	Pt-SPEs was covered COHCFe	CV		Seized samples	Balbino et al., 2016

*RGO-Pd, Graphene-palladium-hybrid-modified glassy carbon electrode; EGO-modified SPE, screen-printed electrode modified by a graphene oxide coating; DPV, Differential pulse voltammetry; IL/NiO/CNTCPE, ionic liquid modified NiO/CNTs carbon paste electrode; SWV, square wave voltammetry; GNSs, Graphene nanosheets; GC, glassy carbon; DDIAS, DNA-directed immobilization of aptamer sensors; SPCEs, Carbone-based SPEsScreen printed carbon electrodes; MWCNTs-SPEs, Screen printed electrodes modified with multi wall carbon nanotubes; Δ^9^THC, Δ-9-tetrahydrocannabinol; Pt-SPE, platinum screen-printed electrode with CoHCFe, a cobalt hexacyanoferrate film; C-SPEs, carbon screen-printed electrodes; CV, cyclic voltammetry; BDDE, Boron-doped diamond electrode; BIA-SWV, batch-injection analysis system with; GSPE, graphite screen-printed electrode; Graphite screen-printed electrodes modified with graphene (GPH-SPE); PABA, p-Aminobenzoic acid; OPD, o-phenylenediamine; molecularly imprinted polymer nanoparticles (nanoMIPs); MIF, molecularly imprinted film; EPAD, Electrochemical paper analytical device; ZnONRs, zinc oxide nanorods; BDD, boron-doped diamond electrode; GC, glassy carbon electrode; MDMA, 3,4-methylenedioxy-methamphetamine; PMA, para-methoxyamphetamine; SCs, Synthetic cannabinoids; GC, Glassy carbon; CA, Chronoamperometry; MEC, 4-Methylethcathinone; NAMM, N-(4-amino-3-methoxyphenyl)-methansufonamide*.

### Narcotics

**Figure 3:**
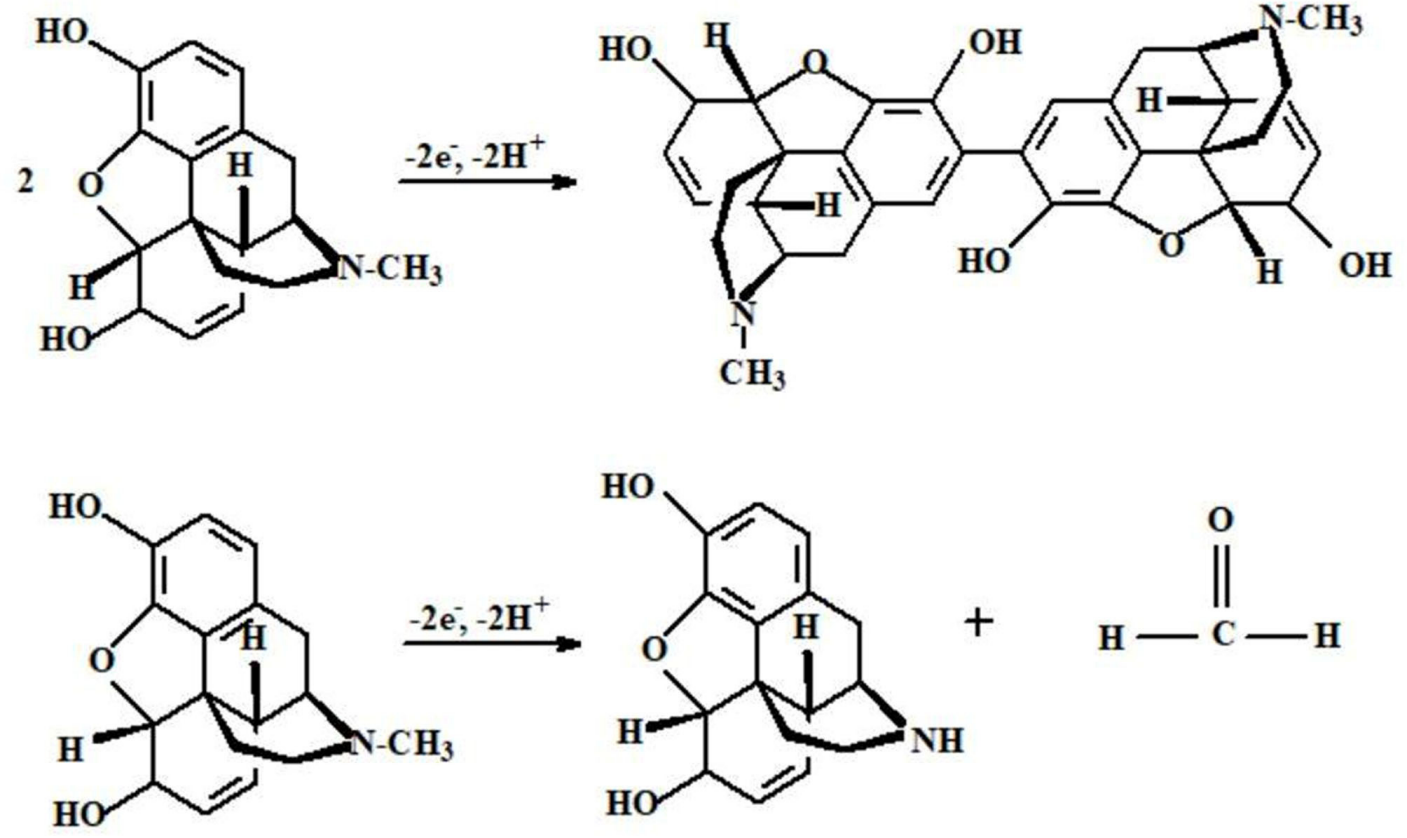
The oxidation mechanism of morphine. Reproduced from Navaee et al. (2012).

Morphine (MO) is a narcotic and a pain-relieving drug, is a highly effective and preferred drug for moderate treatment of severe pain. Two compounds with different structures result following the morphine oxidation process, as can be seen in Figure 3. The first one is related to oxidation of phenolic group, forming pseudomorphine. The second reaction is related to oxidation of the tertiary amine group (Navaee et al., 2012).

Rapid and simultaneous determination of MO in pharmaceutical and illicit samples has remained a great challenge in analytical chemistry. Electrochemical methods, especially voltammetric methods, have been extensively used for opiates detection because these methods are simple and provide improved selectivity (Atta et al., 2014). A novel ionic liquid modified NiO/CNTs carbon paste electrode (IL/NiO/CNTCPE) was investigated by Sanati et al. using square wave voltammetry. This sensing platform had been fabricated by using hydrophilic ionic liquid 1-methyl-3-butylimidazolium chloride as a binder. After the functionalization, these electrodes exhibited a potent and persistent electron mediating behavior and a good peak-to-peak separation of MO and diclofenac. In this case the detection limit was 0.01 μmol *L*^−1^. Successful tests were performed in both human urine and pharmaceutical samples with good results (Sanati et al., 2014).

### Stimulants

**Figure 4:**
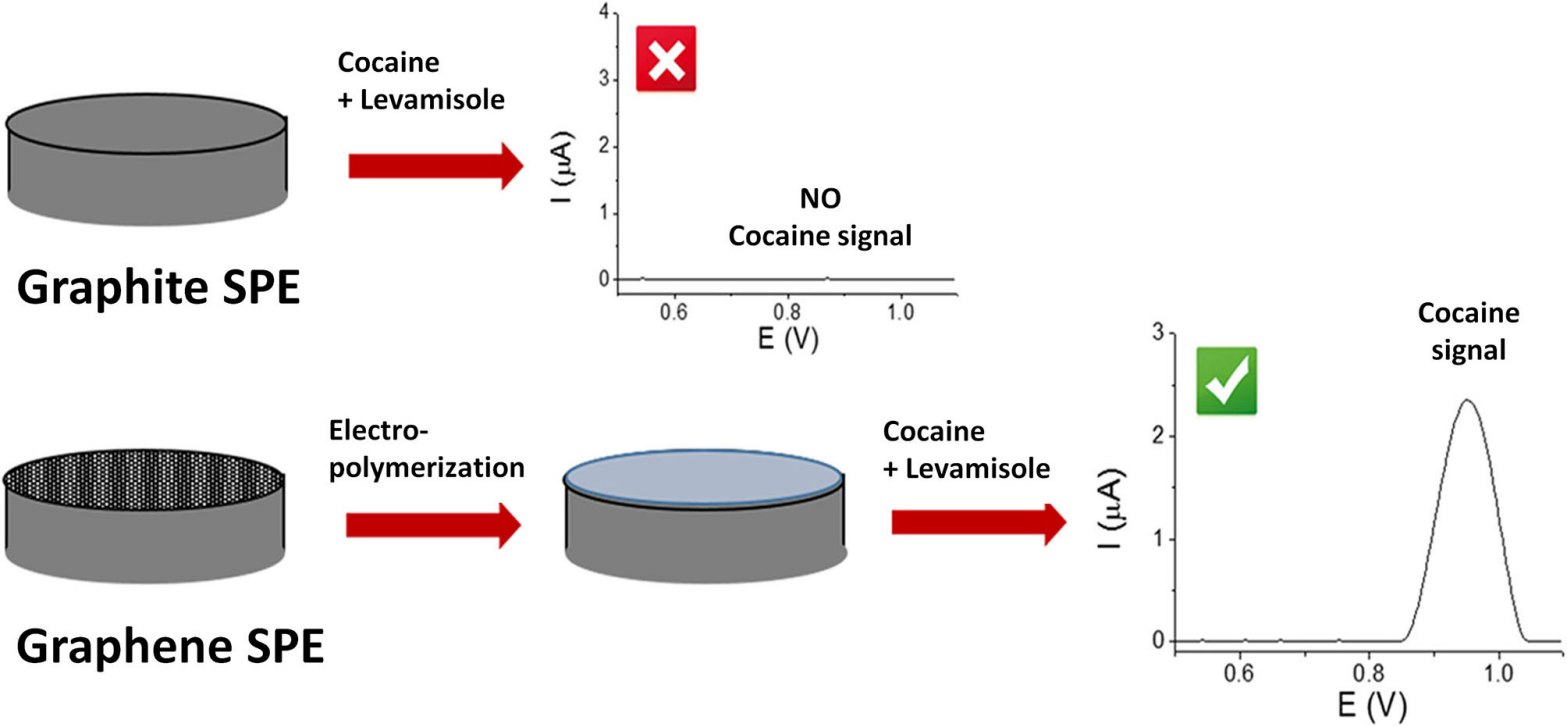
The principle of working of a polymer-based sensor for the detection of cocaine in presence of levamisole. Florea et al. (2018) with permission from Talanta.

Cocaine, an alkaloid extracted from *Erythroxylum coca plant*, is one of the most powerful addictive stimulants that especially affects the brain. It is well known that cocaine is commonly sold containing several other substances. Accurate drug detection is of utmost importance for fighting against drug abuse. These substances are intentionally added to mimic the effects, to dilute the active drug and increase the profits (Fiorentin et al., 2019). The adulterants and cutting agents detection is usually quantified using spectrophotometric or chromatographic methods, which involve high cost instrumentation and impossibilities for on-site detection (Freitas et al., 2017). Recently, the use of electrochemical methods for the detection of adulterants or cutting agents has been reported. For example, in a study developed by L. Asturias-Arribas et al., it was shown that by modification of disposable carbon sensors with carbon nanotubes cocaine could be detected in the same sample with codeine, paracetamol or caffeine without any signal influences (Asturias-Arribas et al., 2014). In another case, Freitas et.al. implemented a sensor for cocaine detection in the presence of benzocaine, caffeine, lidocaine, phenacetine, paracetamol and procaine from a seized sample through a bath-injection analysis system and with square-wave voltammetry. Cocaine and the cutting agents were electrochemically oxidized on a boron-doped diamond electrode (BDDE), resulting in a unique voltammetric profile (Freitas et al., 2017). A new method for the detection of cocaine in the presence of adulterant levamisole on a graphite-based SPE platform through a polymer electrodeposition process was also discovered by Florea et al. (Figure 4). Aminobenzoic acid and *o*-phenylene diamine were electrodeposited onto graphite SPE. This aided to avoid the adsorption of levamisole onto the electrodes which leads to a suppression of the cocaine signal, allowing the simultaneous detection of both cocaine and levamisole (Florea et al., 2018).

Another established class of stimulants is represented by cathinone. The most important synthetic cathinone-based “legal highs” are methcathinone and its derivate, mephedrone, which are structurally related to the natural stimulant, cathinone and possesses a pharmacological similarity to the phenethylamine class of psychoactives (Smith et al., 2013). For cathinone detection a disposable simultaneous electrochemical sensor array based on MIF (molecularly imprinted film) with a SPE has been developed by Zang et al. For the MIF implementation, methcathinone and cathinone were used as templates and pyrrole as the monomer. MIF was synthesized by electro-polymerization. Through the N*H*_2_-graphene (NG) the conductivity of the SPE was improved. Thus, with these modifications, the limits of detection for methcathinone and cathinone were going down to 0.02 and 0.059 nmol *mL*^−1^ respectively. Tests in serum samples were also performed proving the potential of the sensor for clinical applications (Zang et al., 2013). Limits of detection for cathinones which were determined in live and post-mortem whole blood samples are presented in Table 2. These are important to keep in mind when developing sensors for the detection of cathinones in blood samples (Sørensen, 2011).

**Table 2:** Limits of detection determined in live and post-mortem whole blood samples (Sørensen, 2011).

**Cathinone**	**LOD (μmol *L*^**−1**^)**	**LOD (μmol *L*^**−1**^)**
	**samples from**	**samples collected**
	**subjects**	**post mortem**
Norephedrine	13.22	17.85
Cathine	13.24	14.56
Ephedrine	16.36	15.15
Pseudoephedrine	17.57	18.18
Cathinone	14.09	20.8
Flephedrone	16.02	17.12
Metcathinone	8.58	12.88
Metylephedrine	13.96	12.84
Methylpseudoephedrine	7.8	8.92
Ethcathinone	8.37	7.85
Methylone	4.83	5.31
Methedrone	2.61	4.35
Mephedrone	3.95	4.51
Butylone	4.07	4.07
Amfepramone	2.43	3.41

The amphetamine-type stimulants (ATSs) are an important drug group and mainly contain amphetamine and methamphetamine. Aside from this, other compounds are included in this class, such as fenethylline, ephedrine, pseudoephedrine, methylphenidate, and 3,4-methylenedioxymethamphetamine. Globally, the consumption of ATSs is growing every day, so sensitive and sensible analytical methods for their detection are needed. Narang et al. reported an electrochemical analytical device (EPAD) for the detection of MDMA which is mainly use as a recreational drug. The working electrode of EPAD contains zinc oxid nanorods (ZnONRs). Tests were performed at pH 7 in a concentration range between 1 μmol *L*^−1^ and 1,000 μmol *L*^−1^ with a detection limit of 0.1 μmol *L*^−1^ for MDMA (Narang et al., 2018). In another approach Cumba et al. developed a protocol for simultaneous detection of MDMA and para-methoxyamphetamine (PMA) which are usually found in the same sample under the alias “ecstasy,” a very popular drug of abuse. They used SPEs as such and obtained a detection limit of 1.295 μmol *L*^−1^ / 0.227 μmol *mL*^−1^ for MDMA/PMA, 0.207 μmol *L*^−1^ for MDMA, and 0.048 μmol *L*^−1^ for PMA (Cumba et al., 2016).

### Cannabinoids

Balbino et al. discovered a new voltammetric method for ^9^-THC detection that was also successfully applied for target analyte detection from seized samples. This approach was performed in N-N dimethylformamide/water (9:1 V/V), using 0.1 mol *L*^−1^ tetrabutylammonium tetrafluoroborate (TBATFB) 0.1 mol *L*^−1^ as the supporting electrolyte and a glassy carbon disk as the working electrode. ^9^-THC was successfully detected in a range between 0.0076 and 0.0359 μmol *L*^−1^ with a detection limit of 0.0001 μmol *L*^−1^ (Balbino et al., 2012).

## Nanomaterials for Sensors Design for the Detection of Drugs of Abuse

### Graphene and Graphene Derivatives

Since 2004, graphene has become the center piece of scientific research for exhaustive domains from the moment it was synthesized by exfoliation of carbon by Geim and Novoselov (2007). Graphene is a 2D carbon-based nanomaterial with a single layer of sp^2^ hybridized carbon atoms and the distance between two carbon atoms is ~1.42 Å. It represents the basic element point in the synthesis of carbon nanotubes and fullerenes. It has a large surface area (2,630 m^2^ g^−1^, twice as high as that of single-walled carbon nanotubes CNTs), high conductivity, good chemical stability, and mechanical strength. Graphene has an electron-rich π-surface, that enables interaction with targeted analytes *via* van der Waals forces, charge transfer, and π-π interactions (Meng et al., 2019).

The sp^2^ hybridized carbon atoms network can be disrupted by the oxygen containing groups such as carboxyls, epoxides and hydroxyls found in graphene oxide (GO). GO can differentiate between the voltammograms of the drugs due to their high electrochemical conductivity and sensitivity allowing simultaneous detection (de Araujo et al., 2018; Panwar et al., 2019; Rawtani et al., 2019). The outstanding electrical properties of graphene can be restored by reduction via different methods such as thermal, chemical or electrical strategies that result in a material with a high amount of C-O bonds known as reduced GO (RGO) (Fakhari et al., 2014).

During the last decade it was used either as pristine graphene or in other forms such as GO, RGO, chemically functionalized or not for the development of electrochemical (bio)sensors. The major reason is that it is often included in various platforms for an enhanced sensitivity of detection by generating an enlarged surface area and electron transfer rate that amplify the detection signal and analytical performances of the (bio)sensor (Cernat et al., 2015, 2016; Beluomini et al., 2019). The surface of graphene interacts with different analytes *via* van der Waals interactions, electron transfer and covalent bonds depending on the functionalities found on the carbon-based material.

The fact that graphenes are commercially available at a reasonable price, the ease of dispersion in aqueous environment and the facile electrochemical treatments are key assets of the material. All the properties described above favor the development of portable electrochemical sensors suitable for decentralized assays that were already reported for biomedical molecules and drug abuse. The (screen) printed electrodes (SPE) based on graphite or even graphene conductive inks enabled fast adsorption kinetics, selectivity and a large binding capacity and in some case even reusability (Couto et al., 2016). In this case the sensor is prepared via a simple method: (1) the deposition of the modifier by drop coating on the graphite-based working surface (graphene, carbon nanotubes (CNT) or fullerenes/metallic nanoparticles (NPs)/polymeric films or (bio)recognition sites, (2) the addition of the sample on the modified electrode, and (3) the electrochemical assessment by differential pulse voltammetry (DPV) or square wave voltammetry (SWV) methods that can be easily implemented on a miniaturized potentiostat.

Starting from graphene nanosheets the morphologies and functionalities became more sophisticated to keep up with the requirements of biomedical/forensic applications regarding the analytical performances.

### Electrochemical Sensors for Drugs of Abuse Detection Based on Graphene

Graphene nanosheets deposited on glassy carbon electrodes (GCE) were employed for the detection of morphine, heroin and noscapine by DPV, individually, in binary combinations or simultaneously. The presence of graphene allowed the detection of the three opiates at reduced overpotentials at micromolar concentration with no pretreatment steps (Navaee et al., 2012). Electrochemically exfoliated GO has conductive properties even in the absence of an electrochemical reduction protocol due to the presence of a higher density of oxidized moieties. The material allowed the detection of morphine with a limit of detection (LOD) of 0.0025 mg *L*^−1^ with a paper-based sensor suitable for the detection of the molecule in real case scenarios (Maccaferri et al., 2019).

Additionally, graphene bearing amino groups were used as a support for the elaboration of a MIP sensor for the detection of methcathinone and cathinone using polypyrrole as monomer. In this case the role of the carbon-based material was to ensure the substrate for the MIP by enhancing the active surface area and the conductivity of the working surface of the graphite printed electrode. This configuration allowed the detection of methcathinone with a LOD of 3.3 pg *mL*^−1^ and cathinone with a LOD of 8.9 pg *mL*^−1^ (Zang et al., 2013). The synthesis of graphene hybrid materials using metallic NPs represents a strategy to improve the electrocatalytical properties of the new platforms. The fast electron transfer between the electrochemical species from the sample and the composite nanomaterial generally results in an amplification of the signal. A positive aspect relies in the large surface of graphene that allows the homogenous deposition of the NPs with a low risk of aggregation. Reduced GO (RGO) and PdNPs generated a hybrid material synthesized *via* microwave irradiation that showed superior catalytical properties toward the electrochemical oxidation of morphine as in the presence of unmodified RGO. The sensor had a LOD of 12.95 nmol *L*^−1^. This fact highlights the effect of Pd on the sensitivity of the method and the discrimination of the electrochemical signal of the target in the presence of other electroactive species commonly found in biological samples such as dopamine, ascorbic, and uric acid (Atta et al., 2014). The association between AuNPs and graphene were an extensively studied approach during the last decade due to the enhancement in the electron transfer rate, an important feature in the development of electrochemical sensors. In this case, AuNPs act as a stimulator to improve the electron transfer with GO improving reaction kinetics considerably compared to pristine GO material (Kumar et al., 2018). The composite platform acted as the support for an aptamer-based sandwich that allowed the detection of cocaine at nanomolar level, a real advantage for the assessment of real samples (Jiang et al., 2012). In another approach, Maccaferri et al. developed a graphene oxide modified screen-printed electrode (GO-SPE) as amperometric sensor for MO determination. By using this platform, they have obtained a higher sensitivity and a limit of detection of 8.77^*^10^−6^ μmol *mL*^−1^. They also have discovered that the electrocatalytic coating is necessary as modifier for the carbon electrode, for resolving the oxidation peak due to MO oxidation despite the uric acid one (Maccaferri et al., 2019).

Graphene can also be made magnetic. Molecular devices, controlled via magnetic forces, have many applications in the development of bioelectronics for biomedical assays. Iron oxide, either Fe_3_O_4_ or Fe_2_O_3_, represents a magnetic nanomaterial with good biocompatibility, low toxicity and stability, properties that recommend it for the development of (bio)sensors in association with other carbon-based nanomaterials. Magnetic graphene yielded high conductivity, surface-to-volume ratio and magnetocrystalline anisotropy. After the functionalization of this new 2D material with labeled aptamers for cocaine and adenosine triphosphate, the platform allowed the detection of analytes within picomolar range. The electrical contact between the biomolecules and the working surface was made with an external magnet that immobilized it on the surface of the transducers (Tang et al., 2011). Similarly, magnetic RGO in association with polyaniline and AuNPs generated a hybrid platform that was further functionalized with a cocaine aptamer. The impedimetric sensor allowed the detection of cocaine from 0.09 to 85 nmol *L*^−1^ with a LOD of 0.029 nmol *L*^−1^ with satisfactory detection from real samples even in the presence of other commonly found compounds such as heroin and caffeine. Magnetic RGO has an increased surface area and facilitates the electron transfer rate, while the presence of the conductive polymer enhances the stability and conductivity of the platform in general by the presence of hydrophilic groups. The catalytic properties are also increased by AuNPs (Hashemi et al., 2017).

### Carbon Nanotubes

#### Properties of Carbon Nanotubes Important for Sensors

Carbon nanotubes (CNTs) represent a very special category of carbon nanomaterials discovered in 1991 by Iijima (1991), and which quickly became one of the most popular research topics, especially in the field of sensors. This fact is mainly due to their special properties such as increased surface area, high thermal stability, electric conductivity, and mechanical strength, as well as their easy and simple functionalization and modification with inorganic and organic compounds or functions.

The intensive use of CNT as electrode surface modifiers is due to their electrocatalytic activity, efficient promotion of charge transfer in redox reactions as well as to their beneficial influence on the sensitivity of electrochemical response, the last one being influenced by the high surface area to-volume ratio of this nanomaterial. Furthermore, it has been evidenced that CNTs present high compatibility with other different nanomaterials and with biological compound such as enzymes (Azevedo et al., 2019; Xie et al., 2019). These features qualify CNTs for electroanalytical determination of target analytes in complex real samples such as biological fluids, and street forensic samples.

However, it should be mentioned that all the special features of CNTs are not enough for high electrochemical detection capacity. Their functionalization is mandatory before the use for the design of electrochemical (bio)sensors, because it aims to reduce the Van der Waals and π*-*π interactions that occur between the different tubes and that can hinder the proper interaction with the target analytes (Zhang et al., 2018; Li et al., 2019).

#### Strategies for Carbon Nanotubes Immobilization at the Electrode Surface

Although all types of CNTs are uniformly ordered, it presents major limitations due to their high hydrophobicity, for example spontaneous coagulation and low solubility in aqueous solutions (Putzbach and Ronkainen, 2013). To solve this shortcoming, CNTs undergo treatment with concentrate solutions of acids. The most commonly used oxidative mixture consists in concentrate sulphuric and nitric acids and the treatment includes refluxing and sonication steps. After this oxidative treatment, defects on the surface of tubes may occur as well as their shortening. An important type of defect that can be produced on both CNT walls and edges consist in the formation of carboxylated sites. These functions are important because they facilitate the CNTs dispersion in aqueous media and their adsorption of chemical immobilization at the electrode as a mono-layer film *via* amidic bond formation with amine functionalities generated at the surface (Kovtyukhova et al., 2003).

There are several studies published to date that have stated the easy functionalization of the surface of CNTs (Rivas et al., 2017; Camilli and Passacantando, 2018; Azevedo et al., 2019; Rasheed et al., 2019). This can be done by forming both covalent and non-covalent bonds with the modifying entities. A suggestive example in this regard is represented by the functionalization of CNTs with organic functional groups, which transforms the outer surface of the tubes to one with strong physical adsorption capacity and with easily controllable surface charge (Azevedo et al., 2019).

The most commonly used carbon nanomaterials for modification of the working electrode surfaces in voltammetry are undoubtedly CNTs. The electrocatalytic properties of CNTs were assigned to their individual configuration, to some metal impurities, to the modification of their surface before being used for electrode functionalization, alone or after integration in various composites. There are several strategies used for the immobilization of CNT at the electrode surface, such as drop coating of the homogeneous suspensions at the electrode followed by solvent evaporation or by the use of CNT-based ink for printing on different planar substrates (Rezaei and Zare, 2008; Trojanowicz, 2016; Hwang et al., 2019), adsorption at the electrode surface (Asturias-Arribas et al., 2014; Trojanowicz, 2016), dispersion in surfactants or polymers (Lin et al., 2004), covalent immobilization after functionalization with different groups (Park et al., 2011) and incorporation in different composites (Gooding et al., 2003; Putzbach and Ronkainen, 2013).

#### Electrochemical Sensors for Drugs of Abuse Detection Based on Carbon Nanotubes

Electrochemical sensors with CNTs have been successfully designed and have found applications in different fields, such as biomedical, pharmaceutical, environmental, food, defense, forensics, and many more (Trojanowicz, 2016).

Electrochemical sensors have found numerous applications in the forensic research field, especially for the direct and indirect detection and analysis of commonly abused drugs. A wide range of electrode platforms have been developed, including ones with CNTs to achieve increase sensitivity and selectivity for targets in complex real matrix (Shaw and Dennany, 2017). Some applications of CNTs in electrochemical sensing of illicit substances, adulterants and precursors are discussed comparatively here, with emphasis on the advantages given by the presence of the nanomaterial on the electrode surface.

An electrochemical sensor for cocaine based on a disposable screen-printed transducer modified with MWCNTs was developed. In this case, the sensor was obtained by the physical immobilization of the MWCNTs, by their simple adsorption. Firstly, the solid nanomaterial was homogenously dispersed in DMF, the dispersion was dropped on the working electrode surface, and then the solvent was evaporated at room temperature. This sensor allowed the selective direct detection of cocaine through a voltammetric procedure even in the presence of three different possible interferences that could be found in street samples, namely codeine, paracetamol and caffeine (Asturias-Arribas et al., 2014).

In another study, linear sweep voltammetry allowed sensitive detection of cocaine in street seized samples by using a glassy carbon electrode modified with MWCNT presenting –COOH groups. The nanomaterial was immobilized at the electrode together with β-cyclodextrin through the bonds formed with the polyaniline film that was previously electrochemically generated at the electrode. This is an example of low cost, rapid and simple strategy for the high reproducible and specific detection of this drug that is very popular among consumers. A LOD of 1.02 μmol *L*^−1^ was reported for cocaine, the obtained results being confirmed with the reference method, namely high-performance liquid chromatography reference method (Garrido et al., 2016).

Morphine was detected using an optimized square wave voltammetry (SWV) method and a carbon paste electrode modified with ionic liquid and an NiO/CNTs composite. The ionic liquid chosen was 1-methyl-3-butylimidazolium chloride, this element has the role of binder for the nanocomposite based on CNTs. It has been observed that this nanocomposite material acts as a catalyst for the electrochemical oxidation of morphine and exhibited a strong and constant electron mediating effect, allowing a clear separation of the oxidation signals corresponding to morphine and diclofenac. A LOD of 0.01 μmol *L*^−1^ was obtained for morphine, the sensor being successfully applied for the detection of morphine ina complex matrix such as human urine and pharmaceutical samples (Sanati et al., 2014).

The same strategy (i.e., the use of carbon paste for the immobilization of the CNTs at the electrode, was applied for the elaboration of an electrochemical sensor for simultaneous detection of morphine and diclofenac). In this case, carbon paste was modified with vinylferrocene-functionalized MWCNTs. Several electrochemical techniques were applied and SWV was chosen for the evaluation of the analytical performances of the sensor. It was observed that the composite electrode presented electrocatalytic effect on the electrochemical oxidation of morphine, by increasing the current intensity, simultaneously with the decrease of the oxidation potential of the target analyte. These properties allowed the morphine signal to be detected from that of diclofenac, and implicitly their simultaneous detection in real samples with a limit of detection of 0.09 mol *L*^−1^ for morphine and 2.0 mol *L*^−1^ diclofenac, respectively (Mokhtari et al., 2012).

The development of nanotechnology has allowed the miniaturization of the electrochemical cells as well as of the devices necessary for their operation. Furthermore, the signal recorded at the electrode can be nowadays transmitted to a Smartphone or other portable device *via* wireless or Bluetooth connection, an important advantage for rapid in field testing. A number of wearable sensors based on CNTs have been developed. Wearable electrochemical sensors have been designed on gloves and rings for illicit substance detection.

Thus, a ring-based electrochemical sensor was designed for the simultaneous, direct, fast and simple detection of ^9^-THC and alcohol in saliva by using SWV and amperometry. The dual sensing ring was able to detect ^9^-THC by SVW and alcohol by amperometry (enzymatic biosensor), with no visible interference and high sensitivity (0.5 μmol *L*^−1^ in ^9^-THC and 0.2 mmol *L*^−1^ alcohol). The developed wearable dual sensor presents great perspectives for in-field roadside screening for illicit substances detection and for alerting wearers about their own intoxication levels before driving (Mishra et al., 2020). CNT presence makes this sensing platform a very versatile one and that can be adapted for the detection of other compounds of interest including other major drugs.

Another example refers to a rapid, on-site detection of drugs of abuse is a high relevance research topic. Synthetic drugs have undergone a rapid development in the last decades, this field having a continuous dynamic. This fact requires forensic researchers to quickly adapt their techniques and devices to new products found on the illicit drugs market. An example of a synthetic compound that has emerged in recent years is fentanyl, a synthetic opioid with very serious implications for all people who come into contact with it (including those who test the confiscated evidence on the street).

A wearable glove-based sensor has been designed for the decentralized electrochemical detection of fentanyl, the sensor being placed on the glove fingertips. In this case, the flexible screen-printed carbon-based electrodes were modified with a mixture of MWCNTs and 4-(3-butyl^−1^-imidazolio)-1-butanesulfonate, which is an ionic liquid at room temperature. The optimized “lab-on-a-glove” sensing system which works according to the strategy “swipe, scan, sense, and alert,” has been successfully applied for the direct oxidation of fentanyl in both liquid and powder samples, a LOD of 10 μmol *L*^−1^ being obtained using square wave voltammetry (Barfidokht et al., 2019).

To summarize the use of CNTs and graphene in the detection of illicit drugs, the most relevant studies in the literature sensors developments based on CNTs and graphene platforms are presented in Table 3.

**Table 3:** Overview of sensors based on graphene and CNTs platforms.

**Nanomaterial**	**Target drug**	**Platform**	**LOD**	**References**
Graphene	Morphine	Electrochemically exfoliated graphene	8.77 nmol L^−1^	Maccaferri et al., 2019
	Cathinone	Graphene/MIP	0.059 pmol mL^−1^	Zang et al., 2013
	Methcathinone		0.02 pmol mL^−1^	
	Cocaine	GO/AuNPs/aptamers	1 nmol L^−1^	Jiang et al., 2012
	Morphine	RGO/Pd	0.012 μmol L^−1^	Atta et al., 2014
	Cocaine	Magnetic graphene	1.5 pmol L^−1^	Tang et al., 2011
	Cocaine	RGO/AuNPs/Polyaniline/aptamer	0.029 nmol L^−1^	Hashemi et al., 2017
CNTs	Cocaine	MWCNTs-SPE	10 μmol L^−1^	Asturias-Arribas et al., 2014
	Cocaine	MWCNTs-COOH/cyclodextrin	1.02 μmol L^−1^	Garrido et al., 2016
	Morphine	NiO/CNTs	0.01 μmol L^−1^	Sanati et al., 2014
	Morphine	MWCNTs/carbon paste	0.09 mol L^−1^	Mokhtari et al., 2012
	Fentanyl	MWCNTs/4-(3-butyl-1-imidazolio)-1-butanesulfonate	10 μmol L^−1^	Barfidokht et al., 2019

*MIP, molecularly imprinted polymer; RGO, reduced graphene oxide; PdNPs, palladium nanoparticles; GO, graphene oxide; AuNPs, gold nanoparticles; SPE, screen printed electrode; MWCNTs, multiwalled carbon nanotubes*.

## Antibodies, Aptamers, and Molecularly Imprinted Polymers for the Detection of Drugs of Abuse

In the case a highly sensitive and selective detection is strived for, (bio)recognition elementssuch as antibodies, aptamers and MIPs can be integrated in the sensor design. A few examples of electrochemical sensors based on these elements are presented below.

Antibodies are widely used for the development of sensors for illicit drugs detection due to their sensitivity and selectivity. Immunosensors offer precise analyte identification in complex matrices, thanks to the highly specific antigen-antibody immunoreaction.

In a recent study published by Eissa et al., they implemented a multiplex immunosensor using screen printed carbon array electrodes modified with gold nanoparticles for detection of MO, Δ^9^-THC and benzoylecgonine. Antibodies against these target molecules were immobilized on eight electrodes in a sensor array simultaneously, and a competitive assay was used for the detection. The detection time for these three electrodes is between 20–40 min. This method has a good selectivity and sensitivity with a detection limit of 1.2 pg mL^−1^ for MO, 7.0 pg mL^−1^ for Δ^9^-THC and 8.0 pg mL^−1^ for benzoylecgonine. The multiplexed immunosensor was used for the detection of drugs from urine samples spiked with these three drugs. Recovery percentages ranged between 88 and 115% (Eissa et al., 2019).

Aptamers are small (usually from 20 to 60 nucleotides) single-stranded RNA or DNA with a specific three-dimensional structure. Aptamers can form complexes with the target protein to inhibit its expression by blocking its activity. Currently, a large number of generated aptamers can bind various targets, from inorganic molecules to large protein complex or entire cells. The main advantages of aptamers are easy modification, high affinity, and good stability, and because of that, they were widely used as recognition biomaterials in biosensors (Zhang and Cao, 2010; Lakhin et al., 2013; Shen et al., 2015).

Aptamers are so widely applicable that new aptamer-related reports are published almost every day. They are also used for the detection of commonly abused drugs, for example a supramolecular aptamer for cocaine detection were developed by Shen et al. In this work they used a supramolecular aptamer, rolling circle amplification (RCA), and multiplex biding of a biotin-streptavidin system. The aptamer fragment were assembled to a supramolecular aptamer which, in the presence of cocaine, conjugates to streptavidin for anchoring of biotinylated circular DNA. They successfully detected cocaine in a concentration range between 2 and 500 nmol *L*^−1^ with a detection limit of 1.3 nmol *L*^−1^ (Shen et al., 2015). In another approach, Yang et al. implemented a novel quantitative community sewage sensor for rapid and cost-effective estimation of cocaine use trends form wastewater. For this study, a thiolated single-stranded DNA (ssDNA) probe was hybridized with aptamer ssDNA in solution, and this step was followed by co-immobilization with 6-mercapto-hexane onto the gold electrodes to control the surface density to effectively bind with cocaine. They detected cocaine in a range between 10 nmol *L*^−1^ to 5 μmol *L*^−1^ and with a LOD of 10 nmol *L*^−1^ (Yang et al., 2016). An example of aptamer-based sensor using a nanocomposite platform (MWCNT/IL/Chit) for the ultra-low (150 pmol *L*^−1^) detection of cocaine was presented by Roushani and Shahdost-Fard (2015b). The approach uses riboflavin (RF) as redox probe to detect cocaine in the linear range from 2 nmol *L*^−1^ to 2.5 μmol *L*^−1^. The AgNPs-functionalized aptamer was introduced to accelerate the electron transfer kinetics involved in the reduction process of RF and to specifically bind the target molecule. Moreover, the same group reported a similar strategy using AuNPs instead and ferricyanide as redox probe for cocaine sensing (Roushani and Shahdost-Fard, 2015a). This aptasensor showed an improved LOD of 100 pmol *L*^−1^ and with a linear range up to 11 μmol *L*^−1^ cocaine. The applicability of the sensor was tested in human blood serum and selectivity was studied against analgesic drugs.

MIPs are biomimetic receptors widely employed as recognition elements in the construction of electrochemical sensors due to their many advantages such as low cost, simplicity in preparation, stability at various temperature and pH and storage in dry state at room temperature for a long time. MIPs are obtained via the molecular imprinting technique which can be done in a covalent or non-covalent fashion and it involves the polymerization of monomers in the presence of the template molecule (the analyte) followed by extraction of the template from the polymer matrix when cavities are left behind which are complementary in size and shape with the template molecule (Florea et al., 2016, 2019b).

**Figure 5:**
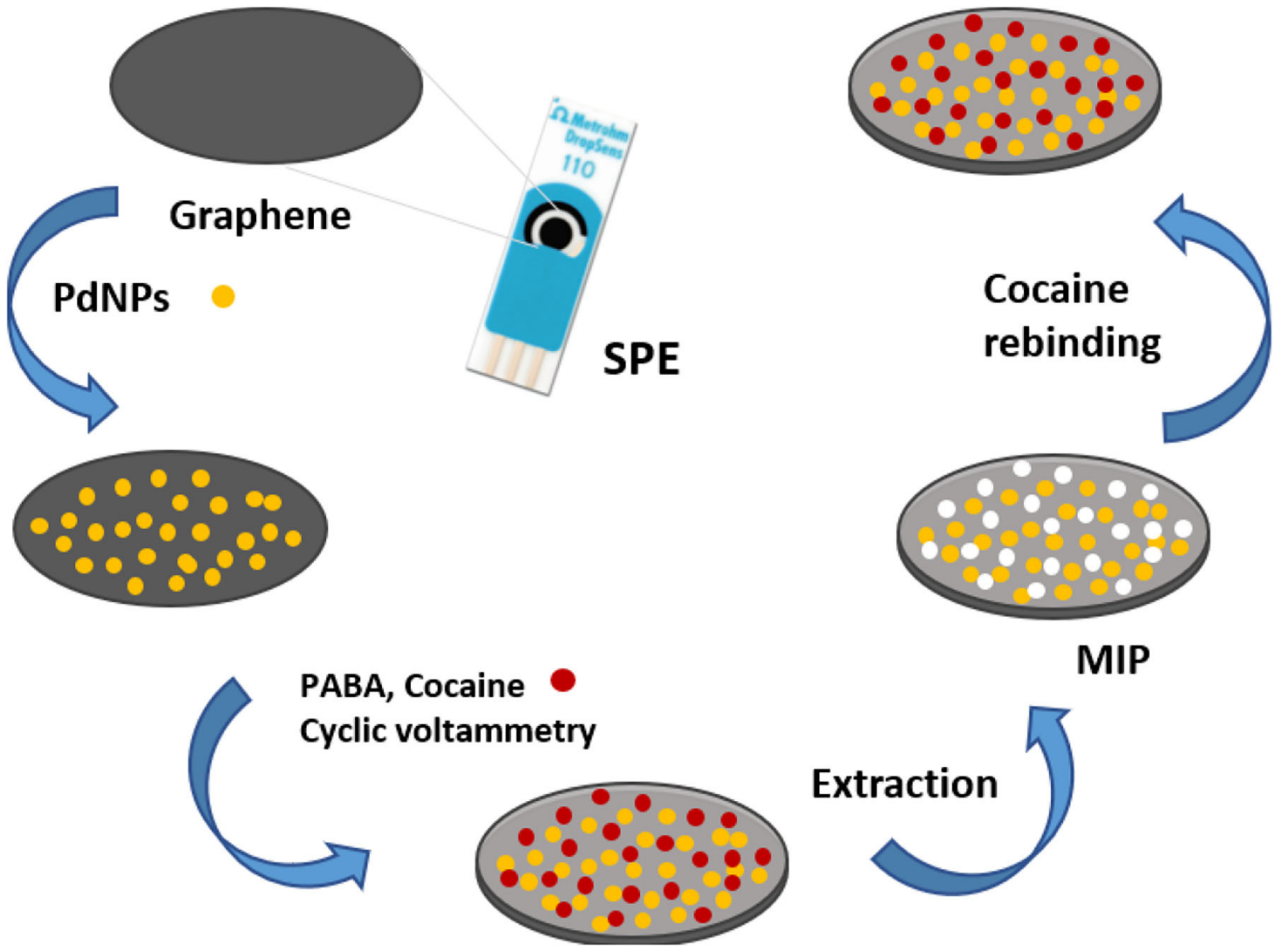
MIP-based sensor fabrication for the detection of cocaine. Reproduced from Florea et al. (2019a) with permission from the Royal Society of Chemistry.

A MIP based sensor for direct detection of cocaine was reported by Florea et al. Palladium nanoparticles were firstly electrodeposited onto graphene SPE for the benefit of enhancing the communication between imprinted sites and electrode and improving their homogenous distribution. Then a MIP layer was synthesized by electropolymerization of *p*-aminobenzoic acid (Figure 5). The appropriate monomer was selected *via* computational modeling to exhibit high binding affinity for cocaine. The signal varied linearly with cocaine concentration in the range of 100–500 μM, with a LOD of 50 μM. The sensor was applied for the detection of cocaine in saliva and river water samples with good recoveries (Florea et al., 2019a).

In another study Smolinska-Kempisty et al. realized a potentiometric sensor for cocaine detection based on molecularly imprinted polymer nanoparticles (nanoMIPs) produced by the solid–phase imprinting method. MIPs are synthetic materials possessing specific binding sites able to recognize a target molecule. MIPs are prepared by co-polymerization of functional monomers and a cross-linker in the presence of a template molecule. In this case the functional monomer used was acrylamide, because it demonstrated a highest yield and a good affinity for the target molecule. The nanoparticles were incorporated in a PVC (polyvinyl chloride) matrix which was then used to prepare an ion-selective membrane integrated with a potentiometric transducer. In this way the nanoMIPs could detect cocaine in a blood serum sample with a limit of quantification of 1 nmol *L*^−1^ (Smolinska-Kempisty et al., 2017).

## Future Perspectives

In the last two decades, the United Nations Office on Drugs and Crime (UNODC) has been focusing on the worldwide safety by hindering the organized crime, corruption, terrorism and drugs threats (United Nations Office on Drugs and Crime). Therefore, in the context of drugs and drugs of abuse, the Laboratory and Scientific Section, Division for Policy Analysis and Public Affairs developed colorimetric testing kits for rapid and simple in field identification of drugs and precursors that are likely to be found in the illicit traffic. A colorimetric test is a presumptive test that indicates the presence or absence of a compound. Colorimetric tests are used in-the-field as a quick and cheap screening method. They are simple, sensitive and the results can be observed visually. However, colorimetric tests often lack specificity. They can be easily influenced by adding certain compounds to the drug causing the test to show a false negative result. The complexation with the cobalt thiocyanate in the cocaine colorimetric test could also take place in the presence of other compounds, causing the test to turn blue, thus leading to a false positive result. Moreover the test is influenced by temperature and the color interpretation is subjective (De Jong et al., 2018b). Therefore, (bio)sensing strategies in detecting drugs offer a clear advantage compared to colorimetric tests, with increased selectivity and comparable analysis time.

Trained drug detection dogs have been used in Australia since the early 2000's. Though, due to the ineffective detection strategy, dogs are more used for the deterrent effect in airports and outdoor music festivals than for detection purposes (Grigg et al., 2018).

The most commonly used strategies for the detection of illicit drugs in biological samples are chromatographic and mass spectrometric methods. However, fast and sensitive determination of drugs of abuse in oral fluid by techniques as Raman, infrared (IR), and nuclear magnetic resonance (NMR), UV-Vis, fluorescence spectroscopy, and surface-enhanced Raman spectroscopy (SERS) are of high interest (D'Elia et al., 2015).

Tremendous progress can be clearly seen in the field of disposable technology for illicit drug detection due to the effort driven by the researchers in this field and law enforcement agencies. For this reason, new portable technology based on instrumental techniques could step forward to traditional forensic laboratories to successfully provide timely and efficient data about illicit threats. Nevertheless, it is believed that their systematic use for preliminary screening would have a great impact on the amount of information that could be collected during investigative processes. Point-of-use instrumental techniques are more often used for screening purposes. In addition, coupling the enhanced properties of nanostructured materials with the sensors in general (Hosu et al., 2019) have allowed the development of simple, fast and low-cost analytical sensing methodologies for illicit drugs.

## Conclusions

One of the biggest challenges of our century is to develop new materials and innovative methods capable to solve humanity's most stringent problems. The detection of abused drugs in field, which could have a positive impact on reducing the drug traffic is one of the challenges. Scientists are making considerable efforts in solving issues related to selectivity and simultaneous detection from seized samples. Nanomaterials and biomimetic elements have the capacity to improve the selectivity, sensitivity and allowed the simultaneous detection. Additionally, electrochemical sensors could be used in field, and could be equipped with disposable sensors avoiding the contaminations.

This review emphasizes the latest technology based on the usage of nanomaterials and approaches based on aptamers and MIPs for the detection of illicit drugs. Despite a few limitations, their outcomes are very promising and could be used as field instruments to be routinely employed by police and policy makers in their investigations.

## Author Contributions

FT wrote sections Examples of (Bio)sensors for Drugs of Abuse and Antibodies, Aptamers, and Molecularly Imprinted Polymers for the Detection of Drugs of Abuse. AF wrote section Introduction, Types of Drugs of Abuse and Their Toxicological Implications, and Antibodies, Aptamers, and Molecularly Imprinted Polymers for the Detection of Drugs of Abuse. AC wrote section Graphene and Graphene Derivatives and Electrochemical Sensors for Drugs of Abuse Detection Based on Graphene. MT wrote section Carbon Nanotubes. OH wrote section Antibodies, Aptamers, and Molecularly Imprinted Polymers for the Detection of Drugs of Abuse, and Future Perspectives. CC did the study design, wrote section Conclusions, and proofread the manuscript. KdW proofread the manuscript. All authors contributed to the article and approved the submitted version.

## Conflict of Interest

The authors declare that the research was conducted in the absence of any commercial or financial relationships that could be construed as a potential conflict of interest.
